# Land use influences stream bacterial communities in lowland tropical watersheds

**DOI:** 10.1038/s41598-021-01193-7

**Published:** 2021-11-05

**Authors:** Karina A. Chavarria, Kristin Saltonstall, Jorge Vinda, Jorge Batista, Megan Lindmark, Robert F. Stallard, Jefferson S. Hall

**Affiliations:** 1 Smithsonian Tropical Research Institute (STRI), Apartado 08-43-0392, Balboa, Ancon, Panama; 2 Department of Hydroscience and Engineering, The University of Iowa, Iowa City, Iowa 52242-1585 USA; 3 ForestGEO, Smithsonian Tropical Research Institute (STRI), Apartado 08-43-0392, Balboa, Ancon, Panama

**Keywords:** Water microbiology, Environmental impact

## Abstract

Land use is known to affect water quality yet the impact it has on aquatic microbial communities in tropical systems is poorly understood. We used 16S metabarcoding to assess the impact of land use on bacterial communities in the water column of four streams in central Panama. Each stream was influenced by a common Neotropical land use: mature forest, secondary forest, silvopasture and traditional cattle pasture. Bacterial community diversity and composition were significantly influenced by nearby land uses. Streams bordered by forests had higher phylogenetic diversity (Faith’s PD) and similar community structure (based on weighted UniFrac distance), whereas the stream surrounded by traditional cattle pasture had lower diversity and unique bacterial communities. The silvopasture stream showed strong seasonal shifts, with communities similar to forested catchments during the wet seasons and cattle pasture during dry seasons. We demonstrate that natural forest regrowth and targeted management, such as maintaining and restoring riparian corridors, benefit stream-water microbiomes in tropical landscapes and can provide a rapid and efficient approach to balancing agricultural activities and water quality protection.

## Introduction

Tropical watersheds are critical to the provision of ecosystem services that sustain livelihoods and ensure the well-being of nearly three billion people^1–4^. Watershed residents and their downstream neighbors rely on freshwater ecosystems for the provision of abundant, clean water for drinking and irrigation, protein, energy, as well as recreation and other cultural ecosystem services^4^. In addition, freshwater streams exchange water, energy, materials and nutrients with the surrounding environment such that water quality, sediment characteristics, and biological communities reflect characteristics from upstream and even downstream environments. Thus diversity and community structure of stream biota are associated with water and sediment^5^ and can be influenced by stream nutritional status, hydrological regimes, and environmental change^6–8^ and may reflect levels of disturbance and degradation^9^.

Freshwater streams receive water from the adjacent land, making them especially vulnerable to the impacts of land-cover change, such as conversion of tropical forest to cattle pasture. Studies have shown that a range of stream properties can vary with a changing landscape, including water temperature, total dissolved solids, and water flow paths^10–12^. Further questions regard the extent to which stream microbial community diversity and structure is maintained or can recover as forests are allowed to regenerate on surrounding land and the tradeoffs made when managing land for livelihoods and other ecosystem services.

Recent studies have highlighted the potential for naturally regenerating tropical secondary forests to rapidly accrue biomass^13^ and sequester vast quantities of carbon such that facilitating and incentivizing these second growth forests can be a useful tool in combating climate change^14,15^. Given that these forests have been shown to recover 80% of their tree species diversity in 50 years^16^ and can recover a significant portion of both saturated hydraulic conductivity^17^ and flow paths in the bio-perturbation zone^18,19^ in less than a decade following cattle removal, promoting growth of secondary forests represents an apparent win–win solution for restoring ecosystem services. However, to date, little is known as to how tropical secondary forests influence water quality and water microbiomes.

Animal husbandry in the form of raising cattle, goats, sheep, horses and other domesticated species cover over one quarter of the land area of Latin America and the Caribbean with clearing forest for cattle production being a major driver of deforestation^4,20^. Given the cultural context of cattle ranching and the human preference for beef and milk, it is unrealistic to expect this to stop. The need to find solutions where some tropical forest ecosystem services can be restored or maintained while the land can also be used for food production^21^ has sparked intense interest in silvopasture systems. Compared to traditional cattle production, silvopasture systems have a number of environmental benefits, including carbon sequestration by trees and restoration and protection of riparian zones by living fences and gallery forests that serve as corridors connecting forest patches across the landscape^22–24^. Along with protecting and planting riparian corridors, a key characteristic of silvopastures is that these have been shown to promote better water quality^23,25^; however, little is known about how these practices impact water microbial communities.

In this study, we assess how secondary forest regrowth and improved agricultural practices influence water quality and stream bacterial communities by comparing stream water from sites where adjacent lands are managed for known ecosystem service tradeoffs. These streams have no known point sources of pollution and are adjacent to four land use types that are common throughout the Neotropics: (a) Mature forest (MF), (b) Young secondary forest (SF), (c) Silvopasture (SP), and (d) Traditional cattle pasture (CP). Our research questions asked: (1) How does land use influence headwater stream bacterial communities? (2) Which microbial taxa (i.e., indicators) are associated with the different land uses? (3) How do traditional water quality metrics correlate with microbial community structure in this tropical landscape? We discuss our results in the context of informing management decisions, including maintaining and/or restoring riparian corridors to protect freshwater microbial community diversity and structure in rapidly changing tropical landscapes that are under intense pressure to provide for local livelihoods and ecosystem services.

## Results

### General characteristics of sampled streams

**Figure 1:**
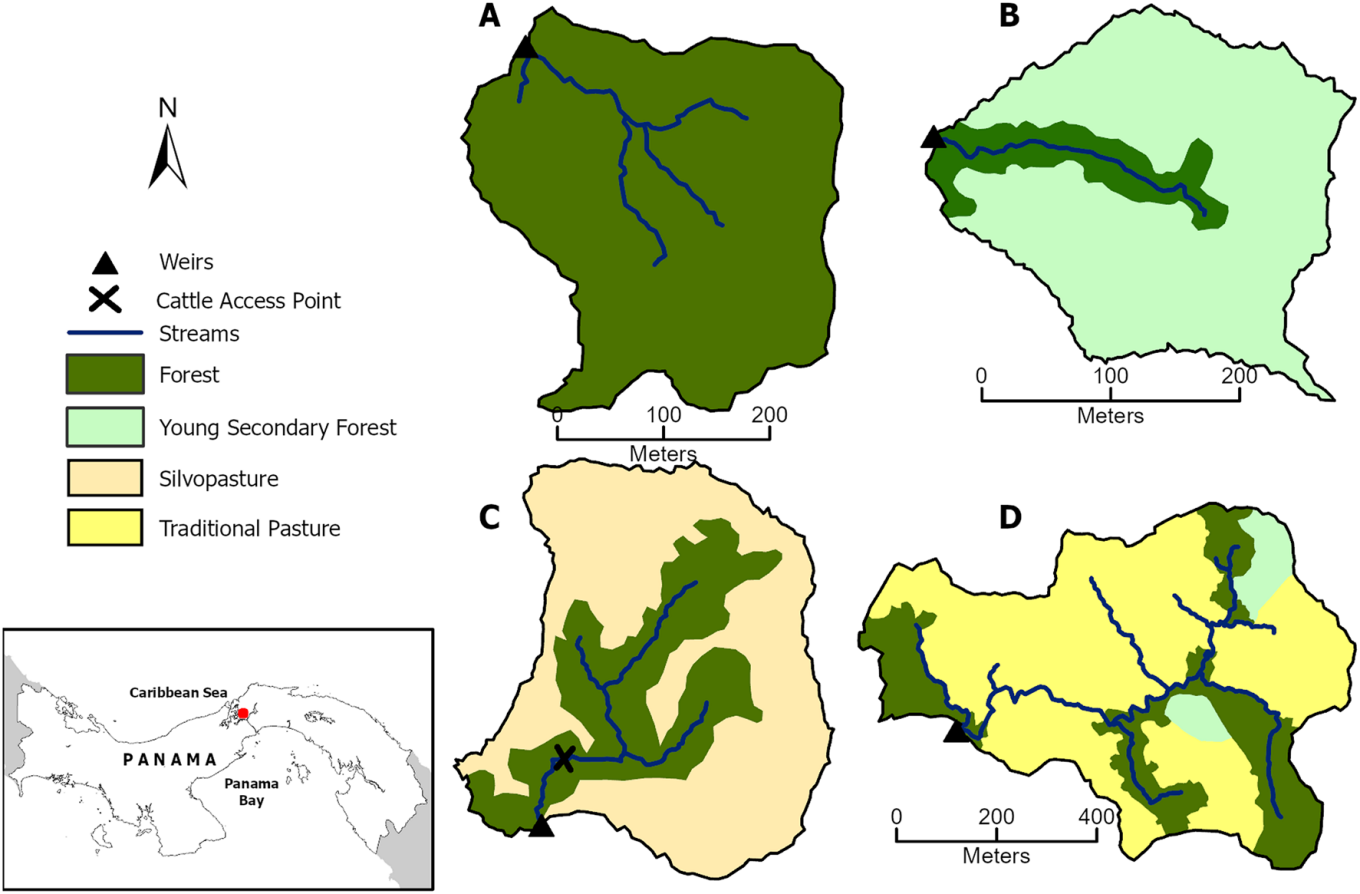
Study sites. Map of Panama indicating location of study sites and schematics of four experimental watersheds in the Agua Salud site showing catchment areas and land use cover: (**A**) Mature Forest (MF); (**B**) Secondary Forest (SF); (**C**) Silvopasture (SP), (**D**) Traditional Cattle Pasture (CP). The maps were generated by Dr. Jefferson S. Hall (co-author) and the Smithsonian Tropical Research Institute team. The maps were generated using ArcGIS Pro from ESRI, version 2.7. All the geographic data are from Agua Salud database, where Smithsonian Tropical Research Institute (STRI) is the owner.

For over 2 years, from June 2017 to August 2019, weekly samples (2 L) were collected at four experimental watersheds within the Agua Salud facility of the Smithsonian Tropical Research Institute, located in the Panama Canal Watershed (PWC) of central Panama (9°13′N, 79°47′W, 330 m above sea level) (Fig. 1). The Agua Salud Project^26^ seeks to understand the ecosystem services produced by tropical forests in a seasonal climate and how they change with land use and climate change. The study area is characterized by a dense network of streams in a landscape of ridge-like hills with steep slopes—a tropical steepland^27^. Mean annual rainfall at the principal meteorological tower is 2700 mm^28^ with an annual precipitation during this study of 2188 mm, 3055 mm and 2339 mm for the years 2017, 2018 and 2019, respectively^29^. The underlying geology and soils of all catchments are similar^26,30^.

Water quality parameters and nutrient concentrations were measured on site and in the laboratory (Supplementary Tables 1 and 2). Concentrations of nitrate, nitrite, ammonia, phosphate, and sulfate were low and similar across sites and seasons, as was pH. Temperature, conductivity, total dissolved solids (TDS), dissolved oxygen (DO), turbidity, total hardness, and total iron concentrations showed more variation (Kruskal–Wallis, *p* < 0.05) (Supplementary Tables 1 and 2). Although DO decreased in all streams during the dry seasons, CP and SP had significantly lower concentrations. Mean water temperatures were significantly lower in the dry season in the MF and SF streams. Stream water total hardness was highest in MF during the dry season and the lowest in CP during the wet seasons (Supplementary Table 1). Total iron concentrations were particularly high in the CP (Supplementary Table 2).

### Stream bacterial diversity and composition

Illumina sequencing produced a total of 3,682,634 high-quality reads that were assigned to 16,985 amplicon sequence variants (ASVs), corresponding to 1210 genera, across 451 water samples from different land-uses (range 2187–23,444 sequences per sample). Sampling across streams was even with 111–114 samples for each land use. Rarefaction curves indicated that sequencing efforts were sufficient to capture the diversity of these communities (Supplementary Fig. S1). Forested streams had more unique ASVs and streams influenced by cattle the fewest (MF = 1894 ASVs, SF = 1815 ASVs, SP, = 1439, ASVs CP = 1442 ASVs). Faith’s phylogenetic diversity of bacterial communities differed among streams (Kruskal–Wallis, *p* < 0.05), with streams in forested catchments (MF and SF) exhibiting the highest diversity while CP had the lowest (Supplementary Fig. S2). Seasonal differences were also observed within each land-use type, with both CP and SP showing seasonal drops in overall diversity during the dry season months of both 2018 and 2019 that were much less pronounced in the forested catchments in 2018. All streams showed a drop in diversity towards the end of the dry season and beginning of the wet season in 2019 (Supplementary Fig. S3).

**Figure 2:**
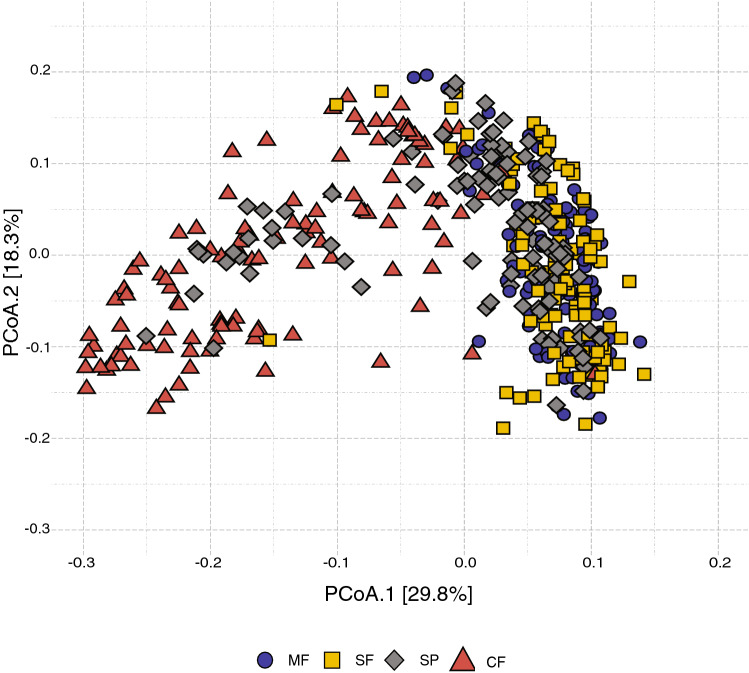
Community composition across sites. Principal coordinates analysis (PCoA) based on weighted UniFrac distances of stream water bacterial communities surrounded by different land uses. Community composition was determined from samples rarefied to 2100 sequences per sample. *MF* Mature Forest, *SF* Secondary Forest, *SP* Silvopasture, *CP* Traditional Cattle Pasture.

Principal Coordinates Analysis (PCoA) using weighted UniFrac distance revealed that communities in CP samples were clearly different from those in the MF and SF but showed some overlap with the SP catchment. Many SP samples also showed overlap with forested sites, while communities in MF and SF clustered closely together (Fig. 2). The first three PCoA axes accounted for ~ 54.6% of the variation, with the CP samples separating from the other land uses mainly along the first axis. Statistical differences were found among all sites and were not due to dispersion among groups (PERMANOVA, *p* < 0.001; ANOSIM, *p* < 0.0001; PERMDISP, *p* > 0.05). Distances based on compositional relationships of community members (i.e., Bray–Curtis, not shown) yielded similar results. PCoA ordination of samples within each land-use type using weighted UniFrac distances revealed seasonal community trends that were particularly apparent in the SP and CP (Supplementary Fig. S4).

**Figure 3:**
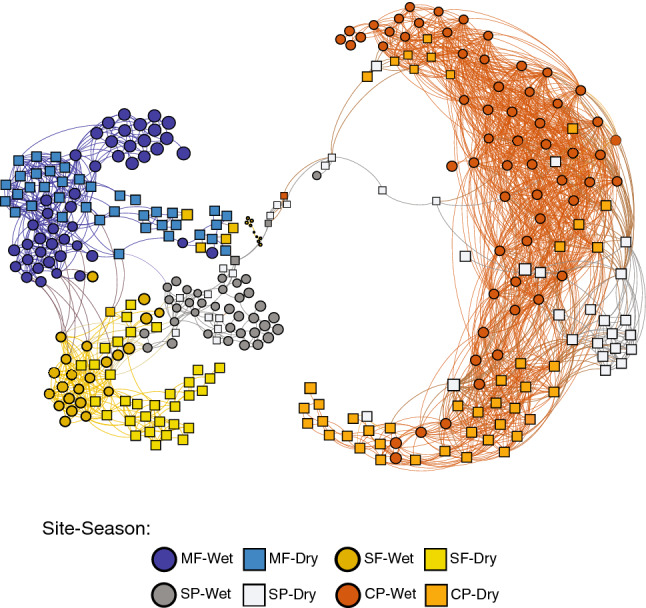
Inferred microbial interaction network. Network of bacterial community structure similarity between stream water samples surrounded by different land use types. The network was built based on co-occurrence of dominant ASVs in these stream bacterial communities. The network was filtered such that connections between ASVs were only kept if they were present in at least 10 samples. *MF* Mature Forest, *SF* Secondary Forest, *SP* Silvopasture, *CP* Traditional Cattle Pasture. Figure was generated using the software R version 3.6.0 (https://cran.r-project.org/), with the packages Phyloseq and igraph, and it was visualized using Gephi Graph Visualization software version 0.9.2 (https://gephi.org/users/).

To explore the effect of land use on microbial interactions, we constructed an inferred microbial interaction network using weighted UniFrac distances (Fig. 3). Communities in the forested streams (MF and SF) clearly clustered together and were distinct from those in the CP, which showed the most variability over the course of our study. The SP stream showed the most dramatic seasonality, where dry season samples clustered with those from CP while wet season samples were more similar to MF and SF. The network had 357 nodes (MF = 87, SF = 79, SP = 85, CP = 106), each representing a distinct sample, 4387 edges connecting these nodes (showing potential similarities), and an average undirected connectivity of 13 and an average clustering coefficient of 0.61.

### Associations between bacterial taxa and land use

There were 19 phyla represented in the dataset, however over 90% of the total community could be attributed to four phyla: Proteobacteria (68.1 ± 1.3%), Bacteroidetes (15.2 ± 1.9%), Epsilonbacteraeota (5.1 ± 4.4%), and Actinobacteria (3.6 ± 2.6%). Proteobacteria, particularly Alpha- and Gamma-proteobacteria, were ubiquitous across streams, although their relative abundance varied across sites and seasons (Table 1)*.* CP, with the lowest relative abundance of Proteobacteria among land-use types, showed a sharp decline in Alphaproteobacteria during the dry seasons and a corresponding increase in the abundance of Bacteroidetes. MF, SF and SP had lower relative abundances of Actinobacteria than CP and higher abundances of Epsilonbacteraeota during all seasons. Throughout our 2-year weekly monitoring, changes in the relative abundance of different top taxa among sites and seasonal variability within sites were noticeable at higher taxonomic levels (Supplementary Fig. S5).

**Table 1:** Seasonal mean relative abundance ± standard deviation of bacterial phyla (> 1% of all ASVs) found in samples from the four streams surrounded by different land uses that were sampled in this study. Within each row, letters indicate significant differences between land uses (*p* < 0.05) based on Kruskal–Wallis and pairwise Wilcox (with Benjamini–Hochberg correction) tests.

Site	Mature forest (MF)		Secondary forest (SF)		Silvopasture (SP)		Cattle pasture (CP)	
Season	Wet	Dry	Wet	Dry	Wet	Dry	Wet	Dry
Phylum—Class	Mean ± SD		Mean ± SD		Mean ± SD		Mean ± SD	
**Proteobacteria**	73.1 ± 0.4^a^	74.1 ± 0.5^a^	75.0 ± 0.4^a^	75.2 ± 0.4^a^	74.7 ± 0.5^a^	74.4 ± 0.6^a^	69.5 ± 0.6^b^	59.2 ± 0.7^c^
Gammaproteobacteria	54.56 ± 0.4	56.3 ± 0.5	54.9 ± 0.4	54.5 ± 0.5	57.9 ± 0.5	58.2 ± 0.7	57.2 ± 0.7	49.6 ± 0.8
Alphaproteobacteria	12.3 ± 0.2	12.4 ± 0.2	12.2 ± 0.2	12.4 ± 0.2	11.2 ± 0.3	13.1 ± 0.4	9.2 ± 0.2	1.7 ± 0.3
Deltaproteobacteria	6.2 ± 0.2	5.5 ± 0.2	8.0 ± 0.2	8.3 ± 0.2	5.6 ± 0.2	3.2 ± 0.2	3.1 ± 0.2	1.9 ± 0.2
**Bacteroidetes**	10.4 ± 0.2^c^	10.7^c^ ± 0.2	8.9 ± 0.2^d^	10.9 ± 0.2^c^	9.3 ± 0.3^c^	12.6 ± 0.8^b^	13.8 ± 0.6^b^	21.3 ± 0.9^a^
Bacteroidia	10.4 ± 0.2	10.7 ± 0.2	8.8 ± 0.1	10.8 ± 0.2	9.3 ± 0.3	12.6 ± 0.8	13.7 ± 0.6	21.3 ± 0.9
**Actinobacteria**	0.6 ± 0.1^d^	0.6 ± 0.1^d^	0.7 ± 0.4^d^	1.0 ± 0.3^d^	1.3 ± 0.5^d^	4.3 ± 0.7^c^	9.2 ± 0.8^b^	13.4 ± 0.8^a^
Actinobacteria	0.3 ± 0.1	0.4 ± 0.1	0.4 ± 0.5	0.8 ± 0.3	1.1 ± 0.5	4.3 ± 0.7	9.1 ± 0.8	13.3 ± 0.8
Thermoleophilia	0.2 ± 0.2	0.1 ± 0.1	0.1 ± 0.1	< 0.1	0.1 ± 0.1	< 0.1	< 0.1	< 0.1
Acidimicrobiia	0.1 ± 0.1	0.1 ± 0.1	0.1 ± 0.1	0.2 ± 0.1	< 0.1	< 0.1	0.1 ± 0.0	< 0.1
**Epsilonbacteraeota**	3.3 ± 2.0^a^	4.3 ± 1.2^a^	2.0 ± 1.0^a^	1.9 ± 1.4^a^	2.9 ± 0.9^a^	2.3 ± 0.7^a^	0.7 ± 0.3^b^	0.6 ± 0.3^b^
Campylobacteria	3.3 ± 2.0	4.3 ± 1.2	2.0 ± 1.0	1.9 ± 1.4	2.9 ± 0.9	2.3 ± 0.7	0.7 ± 0.3	0.6 ± 0.3
Verrucomicrobia	2.8 ± 0.2^a^	2.6 ± 0.2^a^	2.6 ± 0.2^a^	2.8 ± 0.2^a^	2.3 ± 0.3^a^	1.2 ± 0.2^b^	1.7 ± 0.2^a^	2.6 ± 0.4^a^
**Firmicutes**	2.2 ± 0.2	1.7 ± 0.3	1.5 ± 0.1	1.0 ± 0.1	2.9 ± 0.2	1.4 ± 0.1	1.3 ± 0.2	0.5 ± 0.1
Clostridia	1.7 ± 0.2	1.2 ± 0.3	0.9 ± 0.1	0.6 ± 0.1	2.0 ± 0.2	0.9 ± 0.1	0.8 ± 0.2	0.3 ± 0.1
**Planctomycetes**	1.3 ± 0.1^a^	1.4 ± 0.1^a^	1.1 ± 0.1^a^	0.2 ± 0.1^b^	0.6 ± 0.2^b^	0.4 ± 0.1^b^	0.2 ± 0.1^b^	0.2 ± 0.1^b^
Planctomycetacia	0.5 ± 0.1	0.8 ± 0.1	0.2 ± 0.1	0.3 ± 0.1	0.1 ± 0.2	0.1 ± 0.1	0.1 ± 0.1	< 0.1
Acidobacteria	1.3 ± 0.2	0.8 ± 0.1	1.5 ± 0.2	0.9 ± 0.2	0.8 ± 0.2	0.3 ± 0.1	0.7 ± 0.1	0.2 ± 0.1
Patescibacteria	0.6 ± 0.1	0.7 ± 0.1	1.4 ± 0.1	1.4 ± 0.1	1.3 ± 0.2	1.3 ± 0.2	0.5 ± 0.2	0.5 ± 0.1
Omnitrophicaeota	0.6 ± 0.1	0.5 ± 0.2	1.2 ± 0.2	0.9 ± 0.1	0.4 ± 0.2	0.2 ± 0.1	0.6 ± 0.2	0.2 ± 0.1

**Figure 4:**
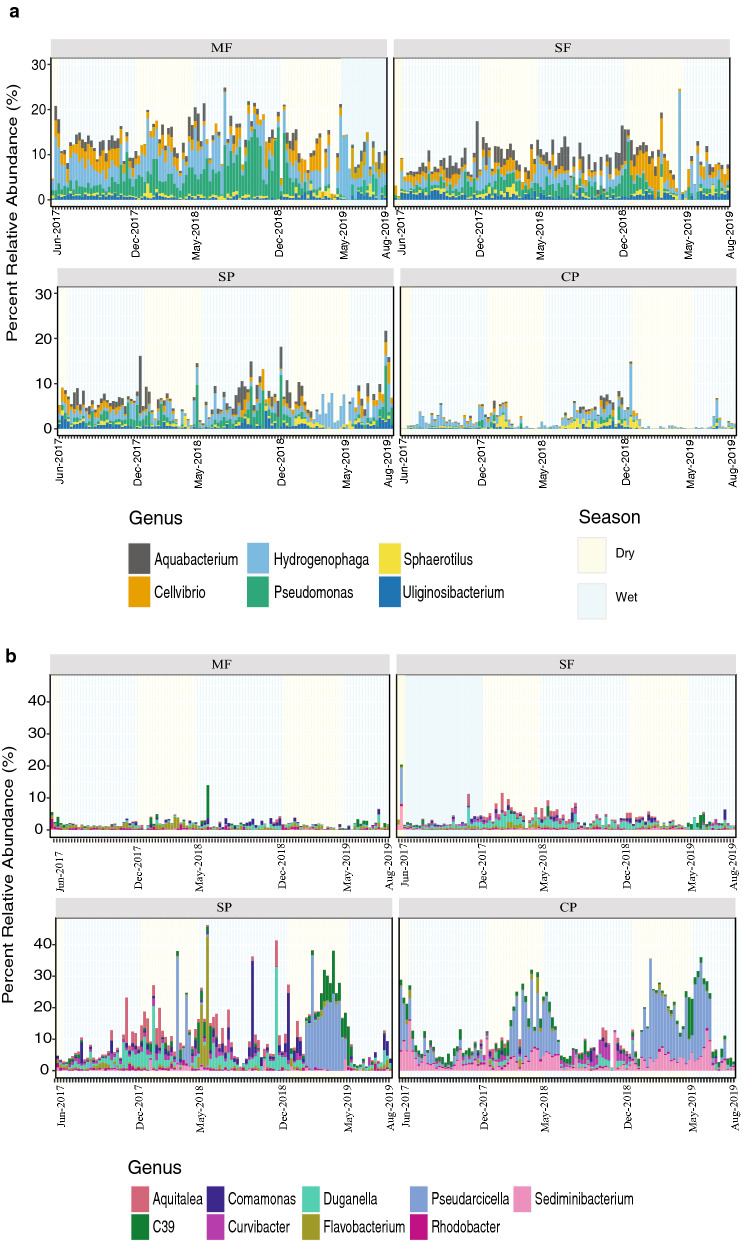
Indicator bacteria. Relative abundance of indicator genera for (**a**) forests and (**b**) pastures across the four land uses based on indicator taxa analysis (Indicspecies). Indicator genera with an overall relative abundance higher than 5% are shown. Colored bars in the plot backgrounds represent seasons. *MF* Mature Forest, *SF* Secondary Forest, *SP* Silvopasture, *CP* Traditonal Cattle Pasture.

Indicator taxon analyses using the R package indicspecies identified 48 indicator genera: 29 for samples collected in forests (MF and SF) and 19 for pastures (CP and SP). When looking only at indicator taxa with relative abundances above 5%, six were observed for forested areas and nine for pastures (Fig. 4). While present at all sites, seasonal patterns of increase and decline of Forest indicator taxa, such as *Hydrogenophaga* (Betaproteobacteria)*, Pseudomonas* (Gammaproteobacteria)*,* and *Cellvibrio* (Gammaproteobacteria) were particularly strong in MF, SF and SP, and they all but disappeared in the CP during the dry seasons. Indicator taxa of streams influenced by cattle pastures showed low relative abundance in the MF and SF, while seasonality was strong in the CP and SP, particularly for *Pseudarcicella* (Bacteroidetes)*, Sediminibacterium* (Bacteroidetes)*, Curvibacter* (Betaproteobacteria)*,* and *Rhodobacter* (Alphaproteobacteria) (Kruskal–Wallis, *p* < 0.05; Fig. 4). Linear discriminant analysis (LefSE) was also used to determine the taxa that described the most variance among samples from the different land-use types (Supplementary Fig. S6). The two forested streams (MF and SF) had many more ASVs showing differential abundance than SP and CP (25 and 26 ASVs vs 7 and 5 ASVs, respectively). Although many of these were only identified to the family or order level, when ASV sequences were further analyzed through the Basic Local Alignment Search Tool (BLAST) we found their closest relatives to be associated with taxa commonly found either in soils or water environments and were also in accordance with our indicator taxon analysis (Fig. 4).

### Associations between bacterial communities and environmental factors

Mantel tests (with 999 permutations) were conducted to look for relationships between bacterial communities (using weighted UniFrac distances) and environment (physicochemical factors) and canonical correspondence analysis was used to illustrate the results (Supplementary Fig. S7a). Bacterial community structure across all sites was positively correlated with pH, dissolved oxygen, turbidity, TDS, total iron and ammonia across all sites; however, all showed “weak” strength of association (*r* < + 0.39). The strongest correlation found among all factors measured was between beta diversity and DO (*p* < 0.001, *r* = + 0.38). Water temperature, nitrite, nitrate, sulfate and phosphate did not correlate with community structure (*p* > 0.05). We used Spearman’s correlation coefficients to assess collinearity and the strength of the correlations between microbial communities and water parameters (Supplementary Fig. S7b). pH was found to be significantly and negatively correlated with several parameters including conductivity and temperature (*p* < 0.05). As expected, conductivity and TDS were significant and positively correlated parameters and also correlated with Turbidity (*p* < 0.001). Conductivity and TDS were also positively correlated with DO (*p* < 0.01).

## Discussion

This study demonstrates that streams adjacent to young naturally regenerated forest growing on abandoned pastures can largely recover bacterial species diversity and community composition as well as measures of water quality after only a decade of cattle removal (Figs. 3, 4; Table 1 and Supplementary Tables 1, 2). We further show that maintaining streamside forest buffers in silvopasture systems helps to protect and restore water quality and stream bacterial communities from the effects of cattle on the landscape, particularly in the wet season. Seasonal differences in bacterial diversity, community structure, and water quality measures in samples from SP and CP point to the detrimental effects of allowing cattle into streams during the dry seasons as bacterial community composition in the SP shifted to resemble that of our traditional cattle pasture during the dry season (Figs. 3 and 4; Supplementary Tables 1 and 2).

### Recovery of stream bacterial communities in secondary forests

Streams influenced by forested sites showed overall higher phylogenetic diversity and more consistent community structure across seasons (Fig. 2 and Supplementary Fig. S2), indicating that these communities are more resilient to temporal and environmental changes. The higher diversity in forested streams also reflects a more robust community that is able to maintain itself throughout the seasons. Samples from SF showed similar levels of diversity as MF (Supplementary Fig. S2), suggesting that these bacterial communities can recover in as little as a decade of forest regrowth in the surrounding landscape, at least in cases such as this where riverine forest was maintained in the past when cattle were present on the landscape but had full access to the stream channel. The communities in SF and MF were also closely related (Fig. 2, 3), yet can be distinguished from each other by a number of taxa that are common in oligotrophic stream systems worldwide, such as members of the Nitrospirae and Pseudomonadales (Gammaproteobacteria) (Supplementary Fig. S6). Many of these taxa have been found in both soils and a variety of aquatic habitats and have been demonstrated to cope well with stressful conditions^31–34^. While seasonal variation in the bacterial communities can be seen in both of these catchments (Fig. 3, Supplementary Figs. S4, S5), their overall diversity remains constant year-round (Supplementary Fig. S2), suggesting that the communities can adapt quickly to conditions, such as changes in stream flow and decreases in DO, associated with seasonality. Our data suggest rapid recovery of dominant forest stream water taxa and the likely ability of the community to adjust to changing conditions when land management permits natural successional processes to occur on the landscape.

### Silvopasture systems can improve water quality

Silvopasture systems are popular land-management systems for their environmental and economic benefits^35,36^. Planting trees in pastures can improve animal health by reducing heat stress^37^ and riverine forests and living fences associated with silvopasture systems further enhance biodiversity values by serving as perches for birds and biological corridors^37,38^. Planting nitrogen-fixing and other tree species can enhance nutrient cycling^39,40^, carbon storage in the soil^3^ and sequestration of carbon in trees^14,15^; however, silvopasture systems may not improve the overall carbon balance as increased stocking can increase methane emissions, particularly in systems where trees and shrub species planted do not directly target improved digestion by cattle. Riparian buffers filter runoff and have been shown to improve stream water quality by reducing the velocity of runoff and promoting infiltration, sediment deposition and nutrient retention^23,41–46^.

Bacterial community diversity and structure for samples from SP showed the most variability, with seasonal associations linking these communities to streams in the other land uses (Figs. 3 and 4, Supplementary Fig. S2). During the dry season months, stream bacterial communities in the SP catchment resembled those of samples taken from CP, with members of the Spirosomaceae family (Bacteroidetes) having a high relative abundance (Supplementary Fig. S5) whereas during the rainy seasons the communities were more similar to forested areas (MF and SF; Fig. 3). Other important taxa driving these differences included *Aquitalea* (Betaproteobacteria), *Pseudarcicella* (Bacteroidetes), and *C39* (Betaproteobacteria) appearing to drive these links to the CP during the dry seasons, while *Aquabacterium* (Betaproteobacteria) and *Pseudomonas* (Gammaproteobacteria) linked the SP community to the forested catchments during the wet seasons (Fig. 4, Supplementary Fig. S5). *Aquitalea*, *Pseudarcicella*, and *C39* are bacteria that have been found in eutrophic water environments^47–49^ suggesting a seasonal influence of cattle in the SP catchment.

Runoff in areas influenced by livestock can influence stream water quality in a variety of ways, including significant sediment and nutrient deposition, fecal contamination inputs and even changes in stream channel morphology^50,51^. We were surprised to find few differences in nutrients, such as nitrogen and phosphorus, between our four catchments given the community changes suggesting fecal inputs in our SP and CP streams. However, Stallard^52^ found similar concentrations of these nutrients in rivers throughout the Panama Canal Watershed that drain the land covers studied here, suggesting that the oligotrophic conditions that we encountered are typical for the region. We did find that samples from CP had higher turbidity and total iron, but lower DO and higher water temperatures during the dry season compared to forested sites (Supplementary Tables 1 and 2). Lower DO concentrations, higher turbidity and water temperatures can result from the concentration of cattle along the streams, which can increase suspended solids and lowering DO as a consequence of trampling along the streambank and excretion of body waste. Higher water temperatures in SP and CP can also be attributed to increased direct sunlight due to less shade near the streams. The higher relative abundance of Bacteroidetes and Actinobacteria in samples from CP (Table 1, Supplementary Fig. S6) combined with lower DO, higher temperatures and lower overall diversity suggests that current cattle management practices in this landscape negatively influence water quality and stream bacterial communities^53,54^. Samples from both the CP and SP had higher abundance of bacteria found in fecal matter, such as *Flavobacterium* (Bacteroidetes), and *Sediminibacetrium* (Bacteroidetes)^55,56^. These genera showed clear seasonal trends, with higher abundance during the dry seasons.

Although total iron concentrations in the CP were found to be higher than the rest of the sites, we think that iron was not the driving factor causing the differences in microbial community composition seen in this catchment. We found ASVs assigned to genera known for iron (II) oxidation like *Gallionella* (Betaproteobacteria) and *Leptothrix* (Betaproteobacteria) as well as iron (III) reduction such as *Geothrix* (Acidobacteria) and *Geobacter* (Deltaproteobacteria; data not shown); however, their relative abundances were low across the dataset and not statistically different among sites. Interestingly, *Rhodobacter* (Alphaproteobacteria), an anaerobic photosynthetic purple bacteria that oxidizes Fe (II) when exposed to light and is found in livestock feces^57^, was found to be an indicator for sites influenced by cattle (Fig. 4). This genus was enriched in both the CP and SP and had very low relative abundances in the forested catchments. However, total iron concentrations were lower in the SP year-round so we do not attribute the higher abundance of this taxa in the CP to higher iron in the water column. Thus, we conclude that although iron concentrations are much higher in the CP stream and there is some signal in the microbial community, total iron is not driving the significant differences in community composition between the CP site and the other sites in this study.

While seasonal shifts in bacterial communities can be seen in all catchments, the SP and CP show the most variation. In our CP catchment, cattle have full access along all stream channels in the catchment and riparian forest covers less that 10% of the catchment area (Fig. 1d). Riparian forest in the SP catchment completely surrounds the stream and is protected with fencing which restricts cattle access to the stream to only a single point (Fig. 1c). During the wet season, cattle visit the stream to drink and return upslope to graze, spending little time near the stream channel. The fenced riverine forest buffer in the SP catchment thus acts as an effective filter, ameliorating the impact of the cattle on the stream channel and helping to preserve water quality^57^, despite nearly two thirds of the catchment surface area being managed as cattle pasture year round. In contrast, during dry season months the cattle tend to spend more time in both the CP and SP streams, where there is more shade, to both avoid the heat and drink. We believe that increased congregation leads to more defecation and disturbance directly in the stream and combined with reduced dry-season flow, drives the reductions in dissolved oxygen, higher water temperatures (Supplementary Table 1), and dramatic seasonal shifts in bacterial community composition (Fig. 3) that we observed in our SP stream. This highlights the benefits of maintaining an extensive riparian forest buffer with fencing but also suggests that installing pumping or other water management systems that keep cattle out of the stream entirely would enhance water quality in stream channels running through livestock farms^58,59^. Providing shade through tree planting or shelters away from stream channels may also reduce cattle congregation in streams in both traditional cattle pasture and silvopasture systems, with corresponding water quality benefits^25^.

Our results demonstrate that land use and forest cover can have significant effects on tropical freshwater bacterial communities. Natural regeneration of tropical forests can rapidly enhance water quality and stream microbial diversity and support the development of robust microbial communities after only a few years of forest growth by improving river bank stability, in-stream vegetation and stream margin vegetation^18,19^. Similarly, the maintenance of riparian forest buffers in silvopasture systems can ameliorate the impacts of cattle pastures and have a positive impact on composition and resilience of aquatic microbiomes. These impacts are most apparent during the wet season when base flow is higher, and cattle are less likely to congregate at stream access points. Indeed, even though microbial communities in our SP catchment showed community shifts indicative of cattle impacts during the dry season, they recovered quickly once wet season conditions returned and became more similar to forested streams. Restoring forest cover and preventing cattle from accessing streams may have important effects on stream health and water quality, with downstream benefits for humans and aquatic fauna alike^58,59^. We recognize that our study is limited to a single example of each land use and that additional study is needed to understand the functional implications of the observed changes in these microbial communities. However, our dataset encompasses 2 years of measurements, spanning three wet seasons and two dry seasons, suggesting that the patterns that we have observed are robust. Management efforts, such as maintaining riparian corridors, limiting livestock access to streams, and permitting forest regrowth, can protect streams from the negative effects of human activities and will likely have cumulative effects, promoting healthier communities and ecosystems downstream and strengthening the resilience of freshwater microbial communities to seasonal and environmental changes in tropical watersheds.

## Materials and methods

### Sampling sites

We selected four streams located in paired experimental watersheds at Agua Salud where stream flow is continually monitored^26^ and land use is not confounded by other upstream uses. These four stream catchments have similar morphologies, soils, underlying geology and rainfall^28^. The streams all show strong seasonal variation in base-streamflow, with reduced flow during the dry season^28^. Each stream is embedded in a different land use: mature forest (MF), young secondary forest (SF), silvopasture (SP) and traditional cattle pasture (CP). The 9.5 ha MF catchment has had continuous forest cover for over 80 years, the earliest dates of our aerial photos. Cattle were removed from the 6.1 ha SF catchment in 2007, 1 year before land acquisition by the Agua Salud Foundation and transfer of management oversight to the Smithsonian Tropical Research Institute. The catchment was a distinct management unit where cattle grazed freely and entered the stream to drink and for shade. Riparian forest covered the entire length of the stream at the time of cattle removal (14% of the SF area) and secondary forest has been allowed to regenerate naturally throughout remaining catchment since 2007. The 42.4 ha CP has been under traditional management since before 1980^28^, with cattle rotated monthly between this and another pasture 5 km distant. Although the 9.4 ha SP management was not established until 2015, the pasture area had been maintained as traditional pasture for at least 40 years prior to planting of improved pasture and fencing of the riparian zone. Both the CP and SP catchments maintain significant tree cover (25% in CP and 32% in SP) but riparian forest makes up < 10% of CP and all 32% of the SP land area. Cattle have complete access to streams along their length in the CP system, whereas stream channels in the SP were fenced in 2015 to limit cattle entrance to a narrow access point for drinking (Fig. 1). Both cattle systems (CP and SP) maintain similar cattle densities at 0.87 and 0.85 head per ha, for CP and SP, respectively. However, although the cattle are in the SP system year-round, in the CP system they are rotated every 30 days between our research catchment and a different farm outside of our research system. Thus, effectively, cattle are only in the CP system 180 days a year following a 30-day rotating schedule.

Water was sampled just above weirs in each of our study catchments. To assess the importance of forest buffers we divided each stream into sections with and without riparian forest buffer on either side of streams during the duration of our study. Stream length was measured from the recorded natural spring where water flow begins to the stream weir. Only the CP system had deforested lengths, which amounted to 50% of the cumulative length of the principal stream and higher order tributaries (Fig. 1).

### Sampling, water quality and nutrient measurements

Weekly 2 L water samples were collected from the MF, SF, SP and CP streams from June 2017 to August 2019 following relevant protocols^60,61^. Most samples were collected between 8:00 A.M. and 12:00 P.M. during baseflow conditions. Samples were transported on ice to the Smithsonian Tropical Research Institute and processed within three hours of collection.

Turbidity, pH, temperature, conductivity, total dissolved solids and dissolved oxygen were measured and recorded on site during each sample collection. Turbidity was measured with a field portable turbidimeter (MicroTPW, HF Scientific Inc., Fort Myers, FL, USA), pH, temperature, conductivity and total dissolved solids were measured with a HANNA portable multiparameter meter (HANNA Instruments, Smithfield, RI, USA), and dissolved oxygen was measured using a portable dissolved oxygen meter (HANNA Instruments, Smithfield, RI, USA). Measurements of nitrite, nitrate, phosphate, sulfate, iron, and ammonia concentrations were measured in the laboratory using the HACH DR 900 Multiparameter Colorimeter (HACH, Vernon Hills, IL, USA). Due to HACH equipment issues, we froze water collected between February 2018 and February 2019, and analyzed them after storage at − 20 °C. Samples were thawed for 2–3 h at 4 °C and analyzed immediately afterwards. To test for changes in nutrient concentrations due to freezing, a subset of 10 samples were analyzed the day of collection as well as being frozen and analyzed after 30 days of storage. Comparisons of these data showed no significant changes in any of the measured nutrients due to freezing. All previous measures were performed following manufacture’s recommendations.

### DNA extraction, PCR amplification and Illumina sequencing

Water samples were stored at 4 °C upon arrival at our laboratory and filtered within 24 h of collection. We filtered one liter of each sample through stacked 1.2 µm and 0.22 µm 47 mm mixed cellulose filters (EDM Millipore, Burlington, MA, USA) using a vacuum pump. Sample filters were then stored at − 20 °C until DNA was extracted.

Isolation of DNA used the DNeasy PowerSoil Kit (QIAGEN, Hilden, Germany) following the manufacturer’s instructions with the following adjustments. To maximize DNA recovery, filters were cut into smaller fragments under sterile conditions prior to the addition of the PowerSoil beads and bead solution, as described in Liao et al., 2015^53^. Isolated DNA was eluted in a final volume of 60 μL. After DNA extraction, all samples were stored at − 20 °C until sequencing libraries were prepared.

Library preparation for amplicon sequencing of the V4 variable region of the 16S rRNA gene was conducted using a two stage PCR protocol. First, the V4 region was amplified using the primer pair 515F (Parada) and 806R (Apprill)^62,63^, modified to include the Illumina sequencing primer on their 5′ end and phased to improve sequencing run quality^64^. PCR reactions included 5 μL Platinum 2 × Mastermix (ThermoFisher), 1 μL of each primer (10 mM) and 2.5 μL of genomic DNA as template (12.5 μL total PCR volume). Thermocycler settings for PCR1 were: initial denaturation at 94 °C for 3 min, 28 cycles of 94 °C for 45 s, 50 °C for 60 s, 72 °C for 90 s and a final elongation at 72 °C for 10 min. PCR reactions were performed in triplicate, verified on a 1.5% agarose gel and pooled after amplification. PCR2 amplifications to add index and remaining Illumina adaptors was carried out with 2.5 μL of pooled PCR product in 12.5 μL reactions with the following program for amplification: 94 °C for 3 min, 8 cycles of 94 °C for 45 s, 50 °C for 60 s, 72 °C for 90 s and a final elongation at 72 °C for 10 min. Products were cleaned and normalized to equal concentrations using Just-a-Plate 96 PCR Purification and Normalization Kit following the manufacture’s recommendations (Charm Biotech, San Diego, CA, USA). The resulting library was further cleaned with AMPure beads and quantified by fluorometric quantification with the HS (High Sensitivity) dsDNA Assay for Qubit (Invitrogen, Carlsbad, CA, USA) and Agilent Bioanalyzer (Agilent, Santa Clara, CA, USA). Paired-end sequencing was performed on a 2 × 250 bp paired end MiSeq run (Illumina, San Diego, CA, USA).

Quantitative Insights Into Microbial Ecology 2 (QIIME2) platform v2020.6 was used for the majority of sequencing data analyses^65^. Primer trimming, sequencing quality control, and forward and reverse read merging were performed using QIIME2’s Cutadapt and DADA2 plugins to produce amplicon sequence variants (ASVs)^65–67^. Taxonomy was assigned using a naïve Bayesian classifier trained on the SILVA 99% sequence similarity database (v32)^68,69^. Sequences were further filtered using BLAST with a 97% confidence. ASVs with low abundance (0.005%) were filtered from the final working ASV table to avoid potential sequencing error^70^. ASVs classified as mitochondria or chloroplast were also removed from further analysis. Downstream analysis was carried out using a rarefied ASV abundance table developed in QIIME2 and was visualized using the R packages Phyloseq and igraph^71,72^.

Alpha diversity metrics (Faith’s Phylogenetic Diversity), beta diversity metrics (Bray and weighted UniFrac), were estimated using QIIME2’s q2-diversity after samples were rarefied to 2100 sequences per sample. Dry season–wet season comparisons were made by grouping samples based on their week of collection; dry seasons included Jan–April from 2018 and 2019, while wet seasons included May–Dec of 2017 2018, and 2019. To assess bacterial community structure and differences among land-use types, both principal coordinate analysis (PCoA) and co-occurrence network was constructed based on the weighted UniFrac distance matrix were visualized. Representative taxa for each land use were identified via indicator species analysis [permutation tests assessing statistical significance of taxa-site group associations based on phi coefficient of association and the indicator value index (IndVal)] using the R package Indicspecies^73^. This analysis determines a parameter ranging from 0 to 1 to assess the quality of the indicators based on the relative abundance and relative frequency across different habitat environments. A genus was considered an indicator taxon if the indicator value for a particular group was > 0.3 and it had a significant p value (*p* < 0.05).

### Statistical analysis

Non-parametric Kruskal–Wallis and pairwise Wilcox tests were use evaluate water quality parameters. Benjamini–Hochberg correction was performed to control for false discovery rates of *P* values. Kruskal–Wallis and pairwise Wilcox tests were also performed to assess differences in ASV abundance and taxa at different ranks in different land-use types and between seasons. To evaluate differences between microbial communities, complementary non-parametric analyses for multivariate data were used: permutational multivariate analysis of variance (PERMANOVA)^74^, and analysis of similarity (ANOSIM)^75^ using weighted UniFrac distance. Permutational analysis of multivariate dispersions (PERMDISP) was used to ensure significant differences were not due to differences in dispersion. Analysis of Composition (ANCOM) and Linear Discriminant Analysis Effect Size (LefSe) were used to identify differentially abundant bacterial ASVs between different land uses^76,77^. A significance threshold was established as *p *$\le \hspace{0.17em}$0.05 with multiple comparisons using 999 permutations. These statistical tests were done in QIIME2 with corresponding q2-diversity and q2-composition plugins. LefSe was computed in R with the microbiomeMarker package^71^.

In addition, correlations between relative abundances of taxa in each sample from each land-use type and all measured environmental factors were calculated using Mantel tests with 999 permutations. Canonical correspondence analysis (CCA) was performed to visualized associations between water parameters and community structure and functional groups.

## Supplementary Information

Supplementary Information.

## Data Availability

Raw sequence files, sample metadata, and QIIME2 derived ASV table are available on Figshare at 10.25573/data.14188418.
