# Supplementary material for: Land use influences stream bacterial communities in lowland tropical watersheds
Source: Sci Rep. 2021 Nov 5;11:21752. doi: 10.1038/s41598-021-01193-7 (PMC8571290; doi:10.1038/s41598-021-01193-7)
Supplement: Supplementary file 1 — Supplementary Information. [file 41598_2021_1193_MOESM1_ESM.pdf]

**Supplementary Information**

# Land use influences stream bacterial communities in lowland tropical watersheds

**Authors**

*Karina A. Chavarria[1*], Kristin Saltonstall[1], Jorge Vinda[1], Jorge Batista[1], Megan Lindmark[2], Robert F. Stallard[1], Jefferson S. Hall[3]*

**Affiliations**

*[1]Smithsonian Tropical Research Institute (STRI), Apartado 08-43-0392 Balboa, Ancon, Panama.*

*[2]Department of Hydroscience and Engineering, The University of Iowa, Iowa City, Iowa 52242-1585, United States.*

*[3]ForestGEO Smithsonian Tropical Research Institute (STRI), Apartado 08-43-0392 Balboa, Ancon, Panama.*

* Corresponding Author: Karina A. Chavarria

*Smithsonian Tropical Research Institute,*

*Amador, Naos, Panama*

*Kari07cha@gmail.com*

**This PDF file includes:**

Supplementary Figures 1 – 7

Supplementary Tables 1 – 2

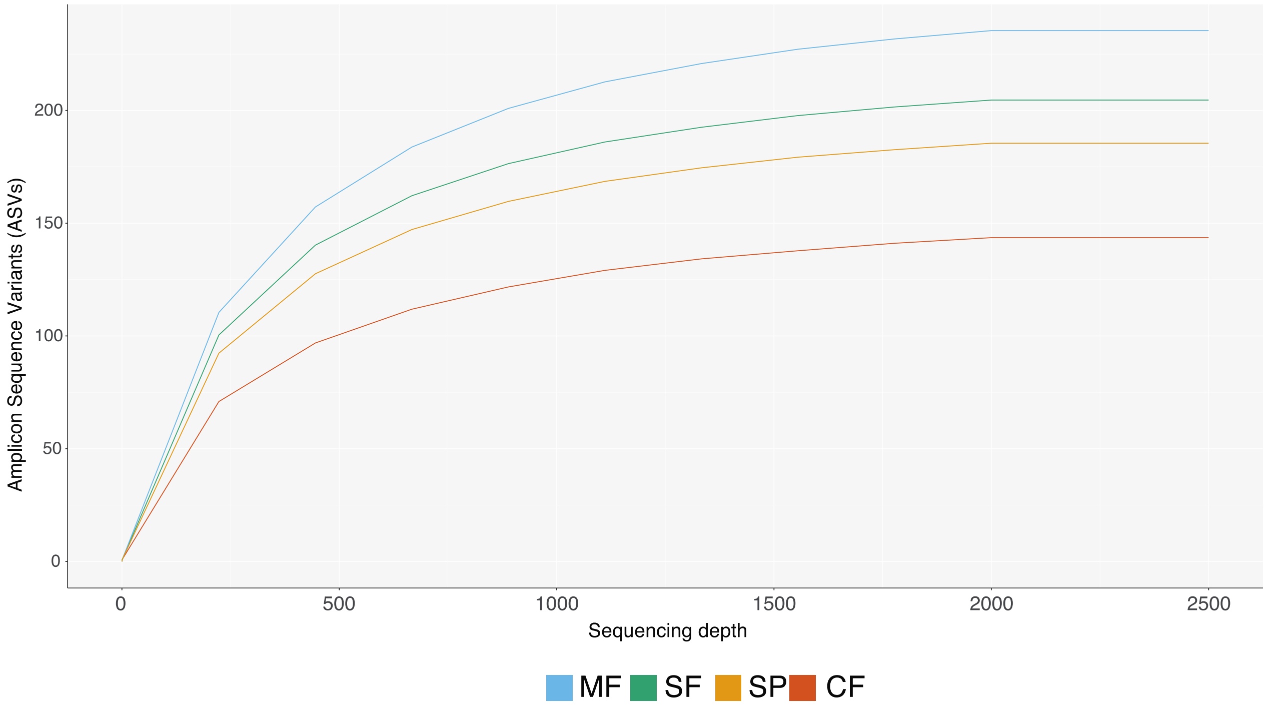


**Supplementary Figure S1. Rarefaction curves of average amplicon sequence variants at each site**. Mature Forest, SF = Secondary Forest, SP = Silvopasture, CP = Traditional Cattle Pasture.

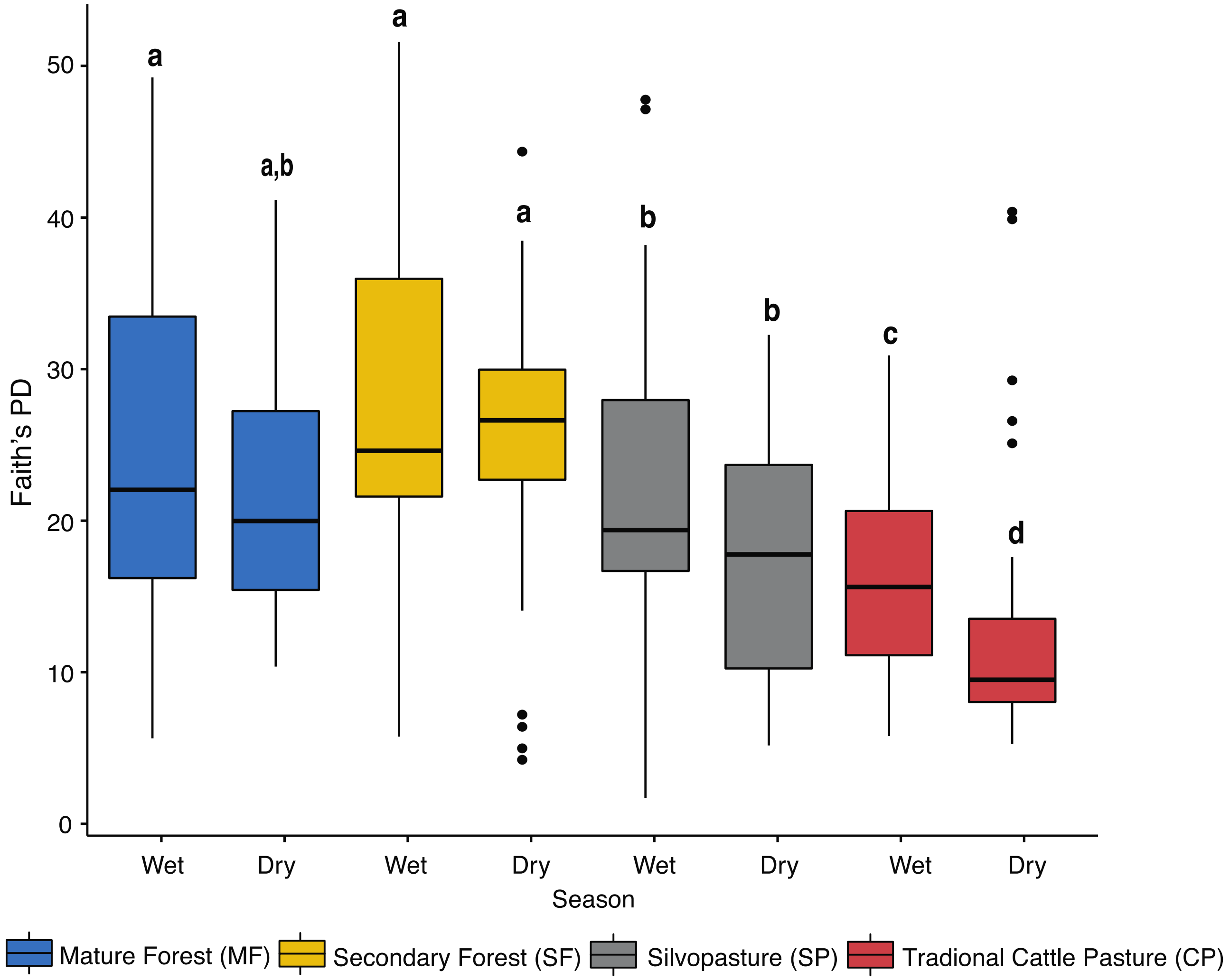


**Supplementary Figure S2. Alpha diversity across sites and seasons**. Faith's Phylogenetic Diversity of stream water in each land use by season. Diversity was calculated using 2100 sequences per water sample. Letters indicate significant differences between land uses ($p < 0.05$) based on Kruskal-Wallis and pairwise Wilcox (with Bonferroni correction) tests. Boxplot center lines show the medians, the upper and lower limits indicate the 25$^{th}$ and 75$^{th}$ percentiles, and the whiskers extend to 150% of the interquartile range. Outlier points are shown by dots.

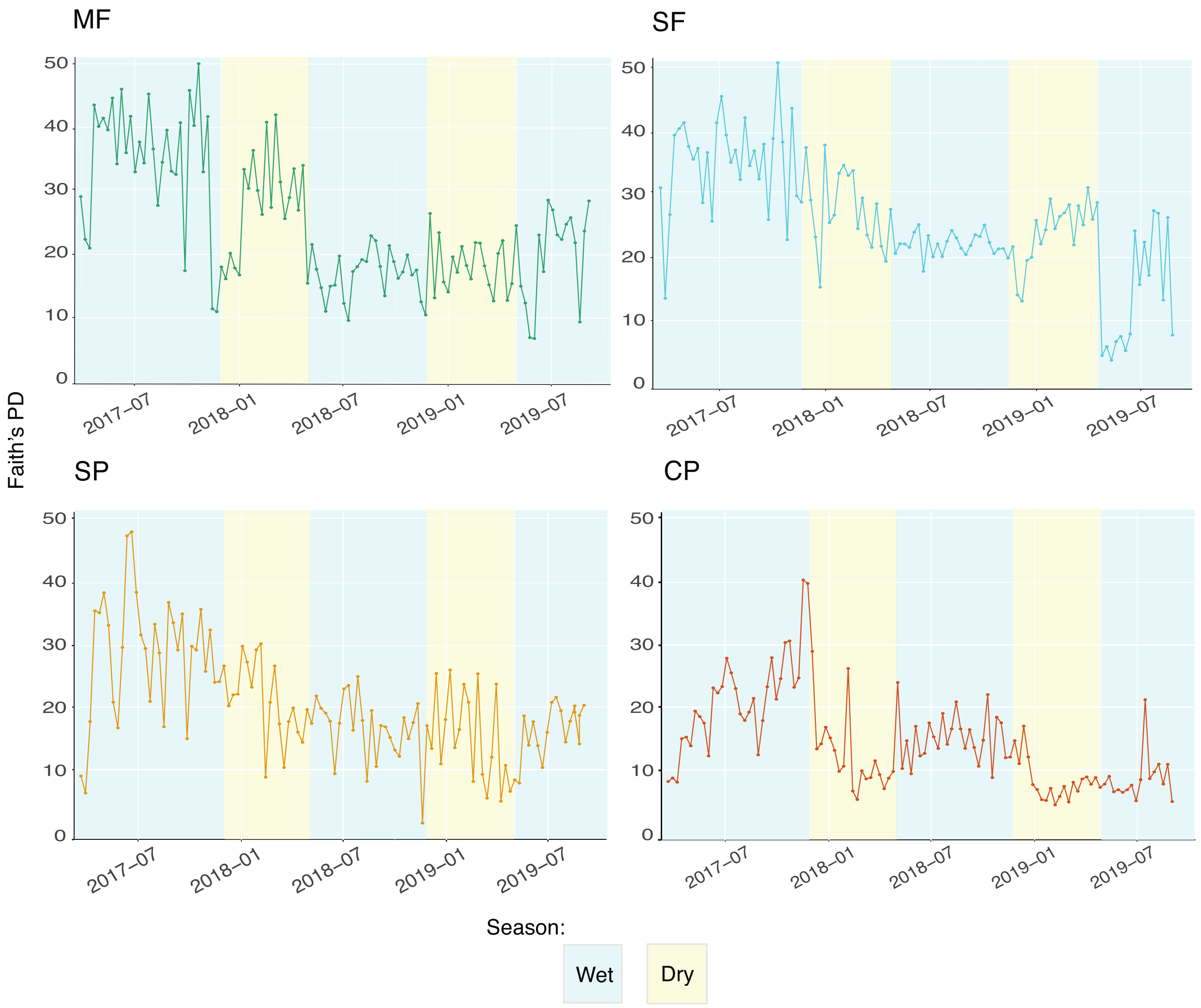


**Supplementary Figure S3**. **Phylogenetic diversity (Faith's PD) across seasons throughout the study time.**

MF = Mature Forest, SF = Secondary Forest, SP = Silvopasture, CP = Traditional Cattle Pasture. Blue background represents wet seasons and yellow background represents dry seasons. Diversity calculations were determined from samples rarefied to 2100 sequences persample.

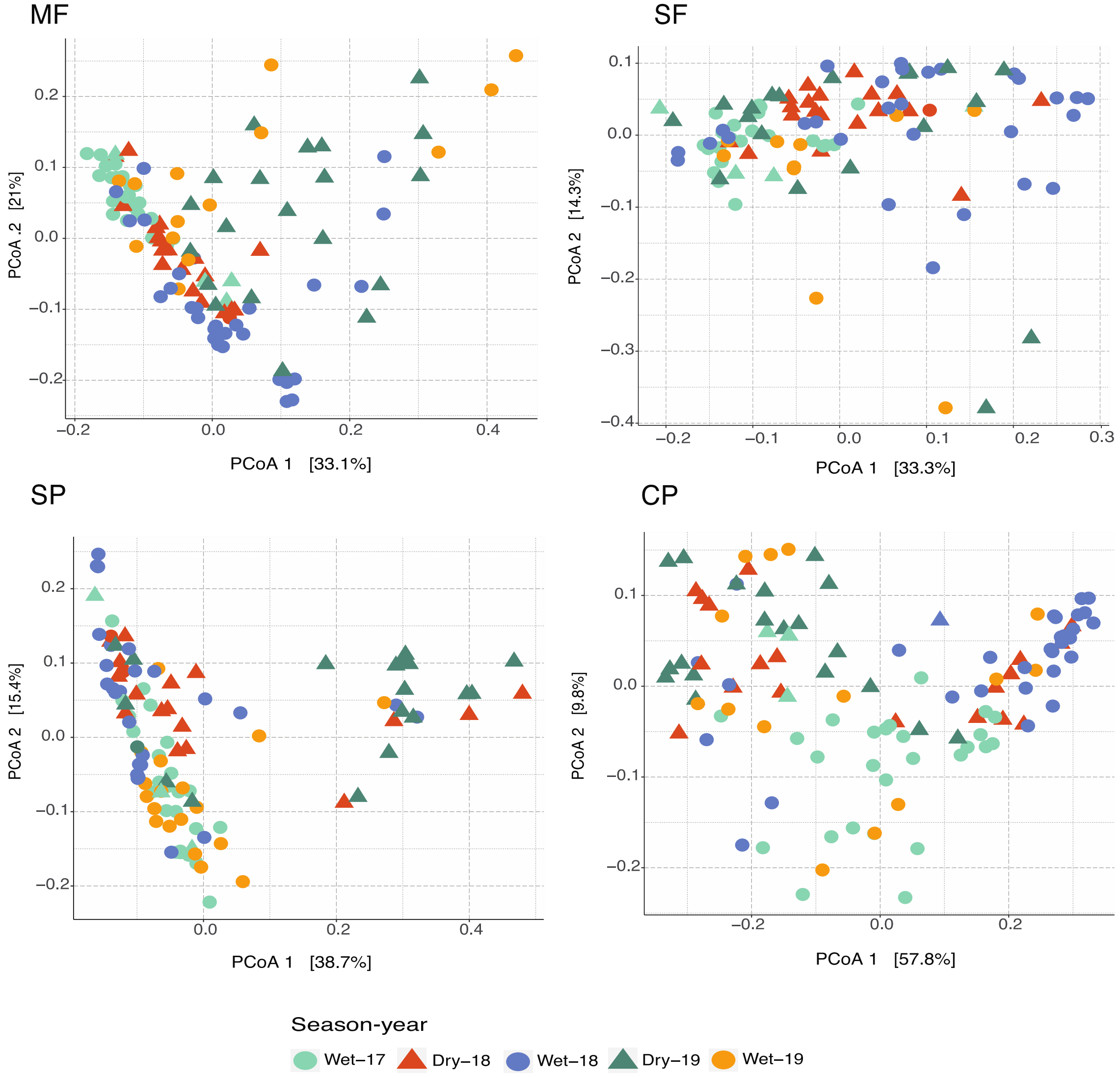


**Supplementary Figure S4. PCoA based on weighted UniFrac distances of samples across seasons.** MF = Mature Forest, SF = Secondary Forest, SP = Silvopasture, CP = Traditional Cattle Pasture. Diversity calculations were determined from samples rarefied to 2100 sequences per sample.

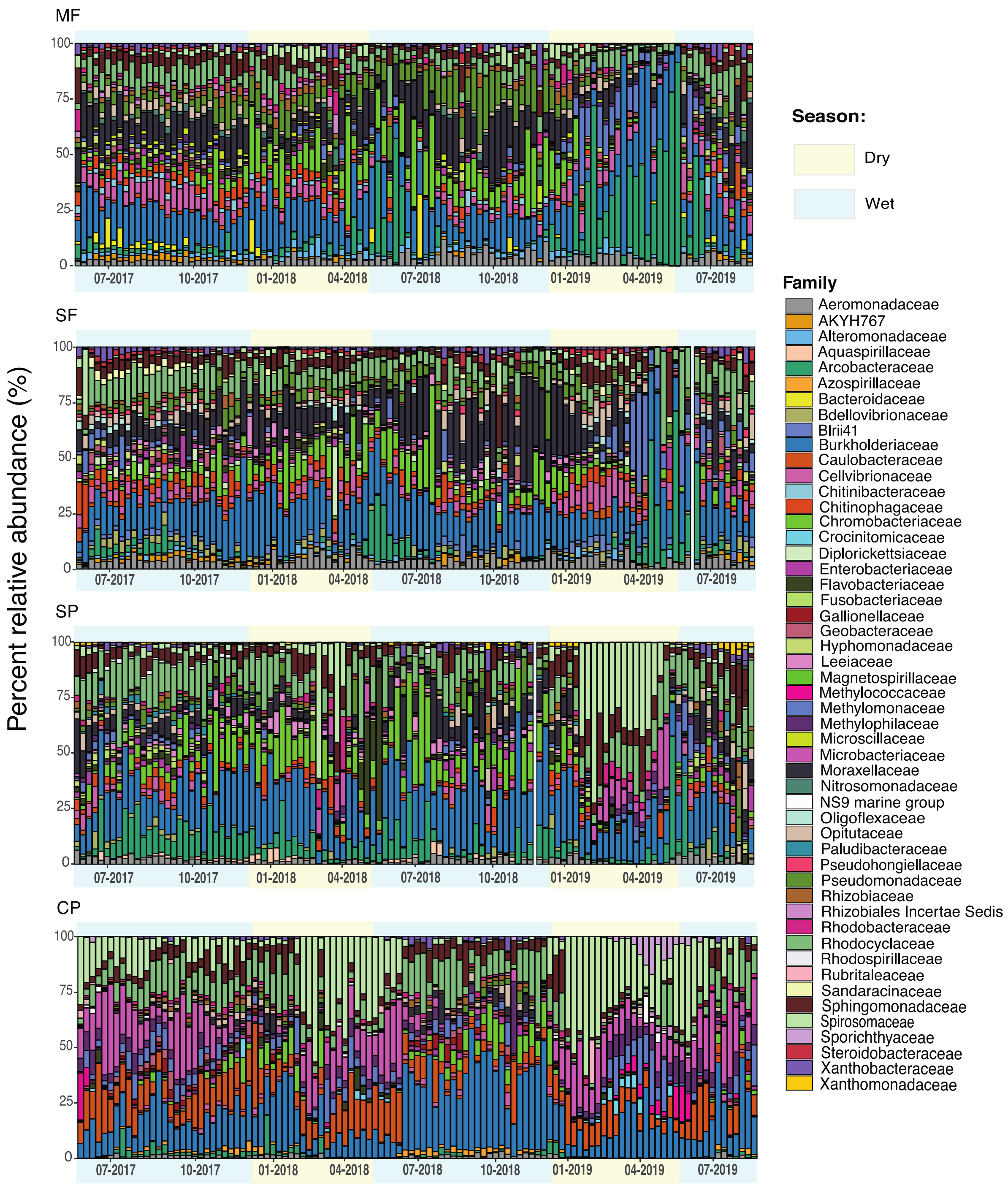


**Supplementary Figure S5. Relative abundance of top 20 families over a two-year period based on weekly sampling**. MF = Mature Forest, SF = Secondary Forest, SP = Silvopasture, CP = Traditional Cattle Pasture. Diversity calculations were determined from samples rarefied to 2100 sequences persample. Figure was generated using the software R version 3.6.0 (https://cran.r-project.org/ ) and the packages Phyloseq and ggplot2. Final formatting was done using Adobe Illustrator 2021 (https://www.adobe.com/products/illustrator.html).

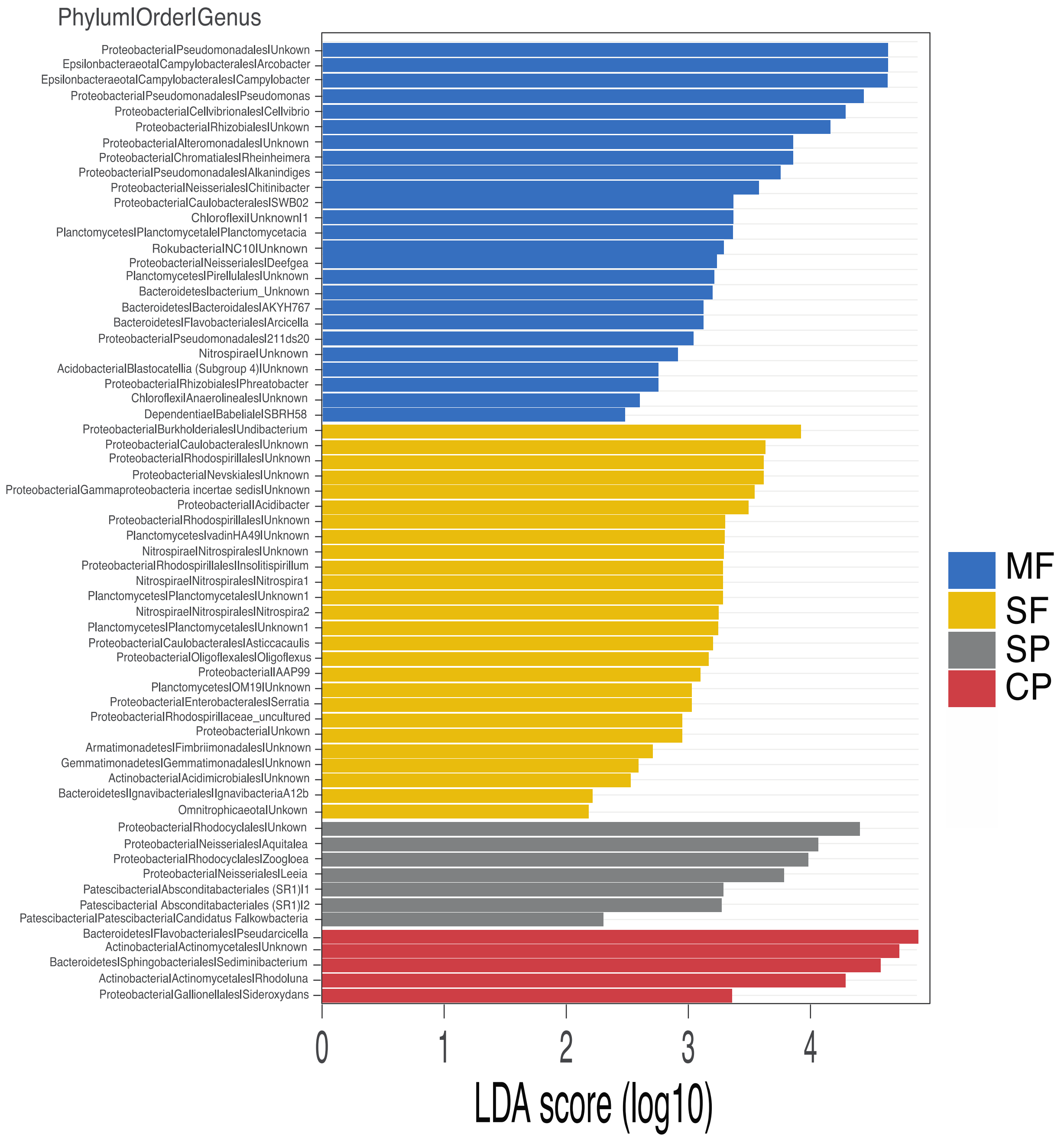


**Supplementary Figure S6. Differentially abundant taxa**. Bacterial taxa identified as differentially abundant between samples from four different land-type uses as analyzed by LefSewith LDA values above 2.0 and $p < 0.05$. MF = Mature Forest, SF = Secondary Forest, SP = Silvopasture, CP = Traditional Cattle Pasture. LDA analysis was determined from samples rarefied to 2100 sequences per sample.

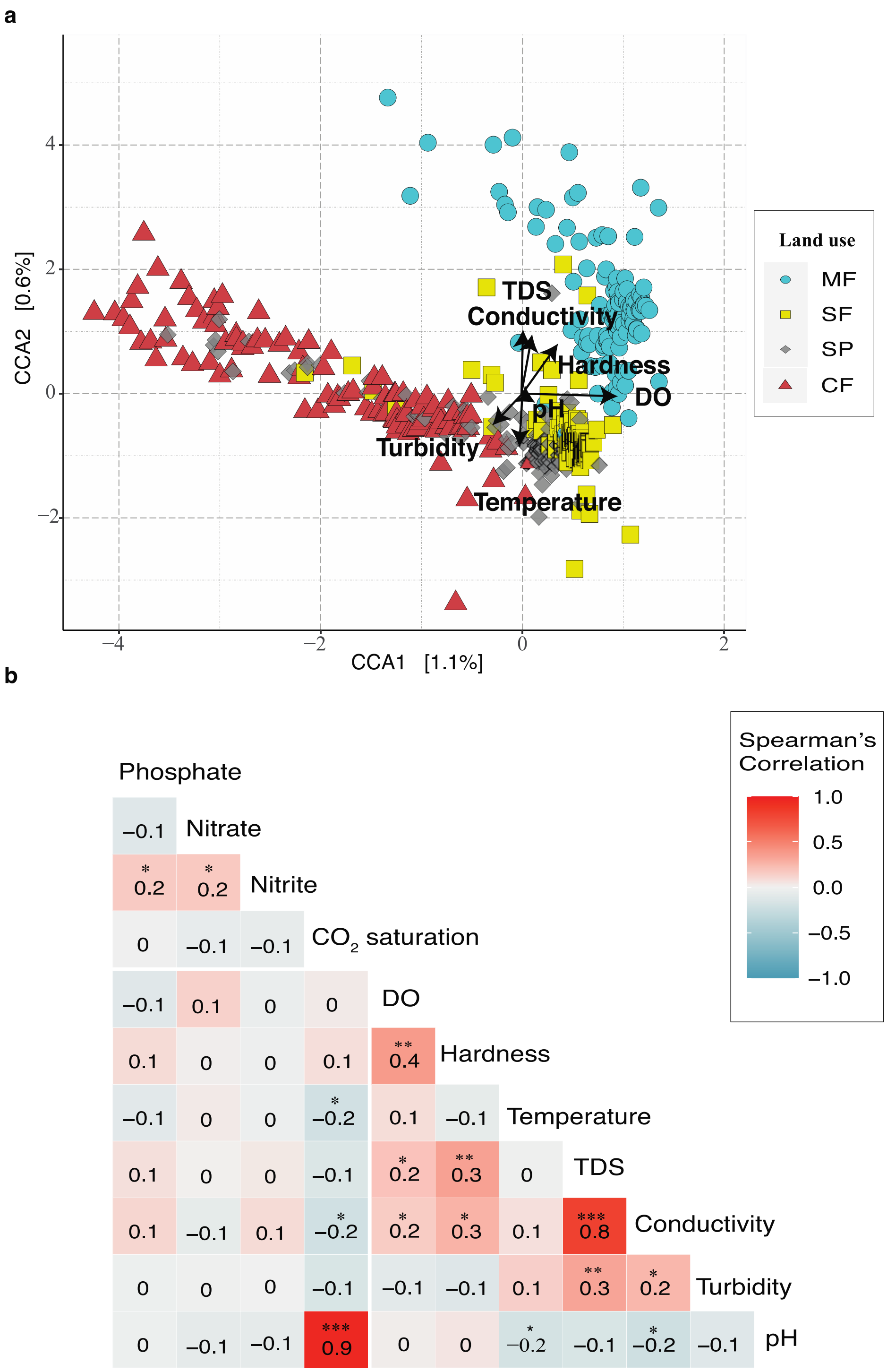


**Supplementary Figure S7. a) Canonical correspondence analysis plot (CCA)** of community composition based on weighted UniFrac distances between samples across land-use type and water quality parameters, and (**b**) **collinearity heatmap of water quality parameters across all catchments**. Arrows refer to environmental conditions. Mature Forest, SF = Secondary Forest, SP = Silvopasture, CP = Traditional Cattle Pasture.

**Supplementary Table 1. Water quality metrics measured in four streams surrounded by different land uses in the Agua Salud research site, Panama**. Values are mean ± standard deviation, according to season. Numbers in parentheses refer to the number of samples tested per site over the course of the study period. Within each row, letters indicate significant differences between land uses ($p < 0.05$) based on Kruskal-Wallis and pairwise Wilcox tests with Benjamini-Hochberg correction.

| Site Location | Mature Forest (MF) | | Secondary Forest (SF) | | Silvopasture (SP) | | Cattle Farm (CF) | |
|---|---|---|---|---|---|---|---|---|
| Area (ha) | 9.49 (n =113) | | 6.08 (n =111) | | 9.36 (n =114) | | 42.39 (n =113) | |
| Season | Wet | Dry | Wet | Dry | Wet | Dry | Wet | Dry |
| | Mean ± SD | | Mean ± SD | | Mean ± SD | | Mean ± SD | |
| pH | 6.9 ± 0.4 | 6.8 ± 0.4 | 7.0 ± 0.4 | 7.0 ± 0.3 | 6.8 ± 0.4 | 6.8 ± 0.4 | 6.80 ± 0.4 | 6.7 ± 0.5 |
| Conductivity (mS/cm) | 0.23 ± 0.1[a] | 0.23 ± 0.05[a] | 0.14 ± 0.07[b,c] | 0.14 ±0.04[b,c] | 0.13 ± 0.07[c] | 0.16 ± 0.1 [c] | 0.17 ± 0.08[b] | 0.22 ± 0.07[a] |
| TDS (mg/L) | 136 ± 34[a] | 150 ± 25[a] | 82 ± 22 [b,c] | 85 ± 17 [b,c] | 85 ± 22 [c] | 87 ± 11[c] | 114 ± 49[b] | 150 ± 53 [a] |
| Temperature (˚C) | 25.5 ± 0.6[a] | 24.9 ± 0.8 [b] | 25.5 ± 0.6[a] | 25.0 ± 0.9[b] | 25.8 ± 0.8[a] | 25.4 ± 1.0[a] | 25.7 ± 0.7[a] | 25.4 ± 0.8[a] |
| Dissolved O2 (mg/L) | 7.3 ± 0.5[a] | 6.3 ± 1.3[b] | 6.7 ± 0.5[b] | 6.1 ± 0.9 [b,c] | 6.2 ± 0.5[c] | 4.8 ± 1.3[d] | 6.0 ± 1.1[b,c] | 4.6 ± 1.3[d] |
| Turbidity (NTU) | 10.4 ± 23.2[b] | 3.7 ± 3.1[c] | 3.5 ± 10.8[d] | 3.5 ± 11.9 [d] | 3.4 ± 4.0 [c,d] | 3.1 ± 4.4[c,d] | 24.6 ± 61.0[a] | 12.4 ± 19.3[a] |
| Hardness (mg/L) | 74 ± 36[a] | 105 ± 52[a] | 49 ± 24[b] | 56.2 ± 27[b] | 46.4 ± 9[b] | 43.5 ± 11[b] | 39 ± 13[c] | 52 ± 14[b] |

**Supplementary Table 2. Water chemistry in four streams surrounded by different land uses in the Agua Salud research site, Panama**. Values are mean ± standard deviation of nutrient measurements, according to season. Numbers in parentheses refer to the number of samples tested per site for each nutrient. Nutrient data measurements include years 2018 and 2019 (Sulfate, Ammonia and Iron measurements only include the year 2019). Within each row, letters indicate significant differences between land uses ($p$ < 0.05)based on Kruskal-Wallis and pairwise Wilcox tests with Benjamini-Hochberg correction.

| **Site Location** | **Mature Forest (MF)** | | **Secondary Forest (SF)** | | **Silvopasture (SP)** | | **Cattle Farm (CF)** | |
|---|---|---|---|---|---|---|---|---|
| **Season** | **Wet** | **Dry** | **Wet** | **Dry** | **Dry** | **Wet** | **Dry** | **Wet** |
| | **Mean ± SD** | | **Mean ± SD** | | **Mean ± SD** | | **Mean ± SD** | |
| **Iron ($\mu$M/L) (n=27)** | 2.2 ± 1.4$^{b}$ | 4.1 ± 1.8$^{b}$ | 2.8 ± 2.4$^{b}$ | 5.8 ± 5.1$^{b}$ | 2.3 ± 1.2$^{b}$ | 8.2 ± 7.9$^{b}$ | 22.1 ± 13.0$^{a}$ | 25.6 ± 11.4$^{a}$ |
| **Nitrite ($\mu$M/L) (n=80)** | 0.2 ± 0.6 | 0.08 ± 0.08 | 0.34 ± 1.17 | 0.06 ± 0.05 | 0.16 ± 0.6 | 0.10 ± 0.09 | 0.16 ± 0.40 | 0.08 ± 0.08 |
| **Nitrate ($\mu$M/L) (n=80)** | 7.4 ± 6.3 | 7.2 ± 5.7 | 11.5 ± 13.6 | 6.6 ± 5.7 | 8.0 ± 6.8 | 11.6 ± 19.1 | 8.6 ± 8.0 | 8.8 ± 7.4 |
| **Phosphate ($\mu$M/L) (n=80)** | 4.9 ± 5.2 | 5.7 ± 5.7 | 4.0 ± 4.0 | 3.7 ± 3.9 | 3.1 ± 3.2 | 4.3 ± 5.6 | 3.7 ± 3.3 | 3.4 ± 2.3 |
| **Sulfate ($\mu$M/L) (n=27)** | 5.2 ± 13.2 | 0.8 ± 2.6 | 9.4 ± 13.4 | 3.5 ± 8.21 | 6.9 ± 12.8 | 2.8 ± 4.6 | 3.78 ± 9.6 | 4.3 ± 6.5 |
| **Ammonia ($\mu$M TN/L) (n=27)** | 1.5 ± 0.4 | 2.4 ± 3.6 | 1.78 ± 0.0 | 2.3 ± 0.9 | 2.7 ± 1.3 | 1.9 ± 4.2 | 3.3 ± 2.2 | 10.0 ± 18.1 |
| **Total coliforms (MPNx$10^4$/100mL) (n=45)** | 2.1 ± 1.3$^{a}$ | 2.2 ± 51.7$^{a}$ | 1.6 ± 1.4$^{b}$ | 1.8 ± 1.2$^{a}$ | 2.1 ± 1.3$^{a}$ | 2.3 ± 1.5$^{a}$ | 3.0 ± 1.5$^{a}$ | 1.2 ± 1.1$^{a,b}$ |
| **E. coli (MPNx$10^2$/100mL) (n=45)** | 3.6 ± 4.0$^{b}$ | 1.9 ± 1.3$^{b}$ | 3.2 ± 2.8$^{b}$ | 5.2 ± 11.0$^{b}$ | 5.5 ± 4.0$^{a}$ | 7.9 ± 10.0$^{a}$ | 10.0 ± 9.1$^{a}$ | 1.7 ± 1.9$^{b}$ |
